# Unusual fungemia caused by non-*marneffei Talaromyces* in an immunocompetent host

**DOI:** 10.1128/asmcr.00096-25

**Published:** 2025-10-15

**Authors:** Eun Jeong Won, Jina Lee, Jae Suk Baek, Seunghwan Seol, Sookja Park, Hyang Burm Lee, Heungsup Sung, Mi-Na Kim

**Affiliations:** 1 Department of Laboratory Medicine, Asan Medical Center, University of Ulsan College of Medicine 37994, Seoul, South Korea; 2 Department of Pediatrics, Asan Medical Center, University of Ulsan College of Medicine 37994, Seoul, South Korea; 3 Department of Agricultural Biological Chemistry, College of Agriculture and Life Sciences, Chonnam National University 34931, Gwangju, South Korea; Rush University Medical Center, Chicago, Illinois, USA

**Keywords:** *Talaromyces tumuli*, fungemia, central line-associated bloodstream infection

## Abstract

**Background:**

*Talaromyces*, a genus phylogenetically related to *Penicillium*, is saprophytic except for *Talaromyces marneffei*. Herein, we describe the first documented case of fungemia caused by *Talaromyces tumuli*.

**Case Summary:**

A case without overt predisposing conditions had suffered from recurrent bacteremia of various causes over the past 3 years and was admitted due to prolonged fever despite antimicrobial therapy this time. Three sets of blood cultures were negative at admission. On hospitalization day (HD) 5, two sets of blood cultures obtained via a subclavian venous catheter (SVC) became positive for hyphae in aerobic vials only with detection time of 67 h, respectively. Subculture of positive blood culture vial to Sabouraud dextrose agar yielded blue-green colonies without production of red pigments. Microscopic examination showed biverticillate *Penicillium*-like conidiophores and species identification using the MALDI Biotyper sirius System (Bruker) resulted in a first-rank match to *Talaromyces funiculosus* with non-reliable identification score of 1.63. Sequencing of the regions of *ITS, BenA, CaM*, and *RPB2* genes identified it as *T. tumuli*. Subsequent blood cultures remained positive for it on six episodes until removal of the SVC on HD 18, despite voriconazole treatment.

**Conclusion:**

This case was the first case of a central venous catheter-associated bloodstream infection caused by *T. tumuli*.

## INTRODUCTION

*Penicillium* species isolated from clinical specimens are often discarded as contaminants (1). Unlike *Penicillium* species, *Talaromyces marneffei* (formerly *Penicillium marneffei*) is a true pathogen causing systemic infections such as fungemia, especially when the patients are in immunocompromised status (2–4). Recently, non-*marneffei Talaromyces* species also have been infrequently reported as emerging opportunistic pathogens in immunocompromised patients (5–8). Here we describe the first case of fungemia caused by *Talaromyces tumuli* in a young woman without a predisposing condition except an indwelling central venous catheter (CVC).

## CASE PRESENTATION

**Fig 1:**
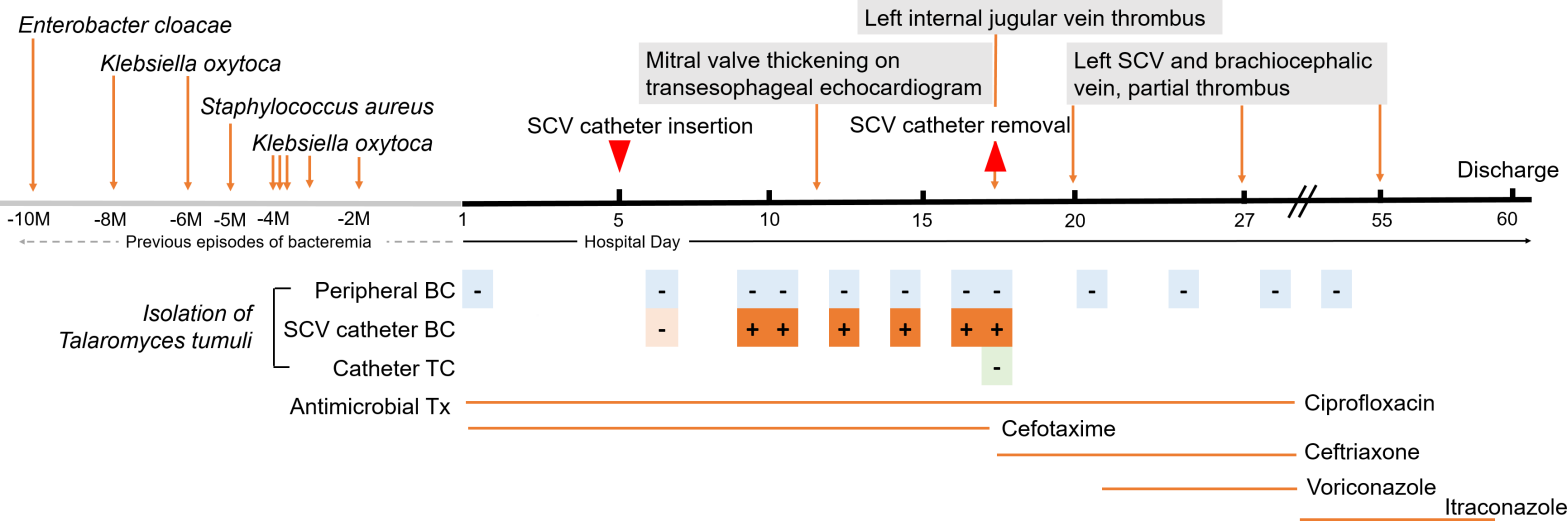
Timeline of medical history in this study. The chronological graph shows the medical history, including transesophageal echocardiography, subclavian vein catheter insertion and removal, blood cultures, catheter tip cultures, and antimicrobial therapy during hospitalization, as well as multiple previous episodes of bacteremia occurring in another hospital. SCV, subclavian vein; M, month; BC, blood culture; TC, tip culture; Tx, therapy; -, no growth; +, growth of *T. tumuli*.

**Fig 2:**
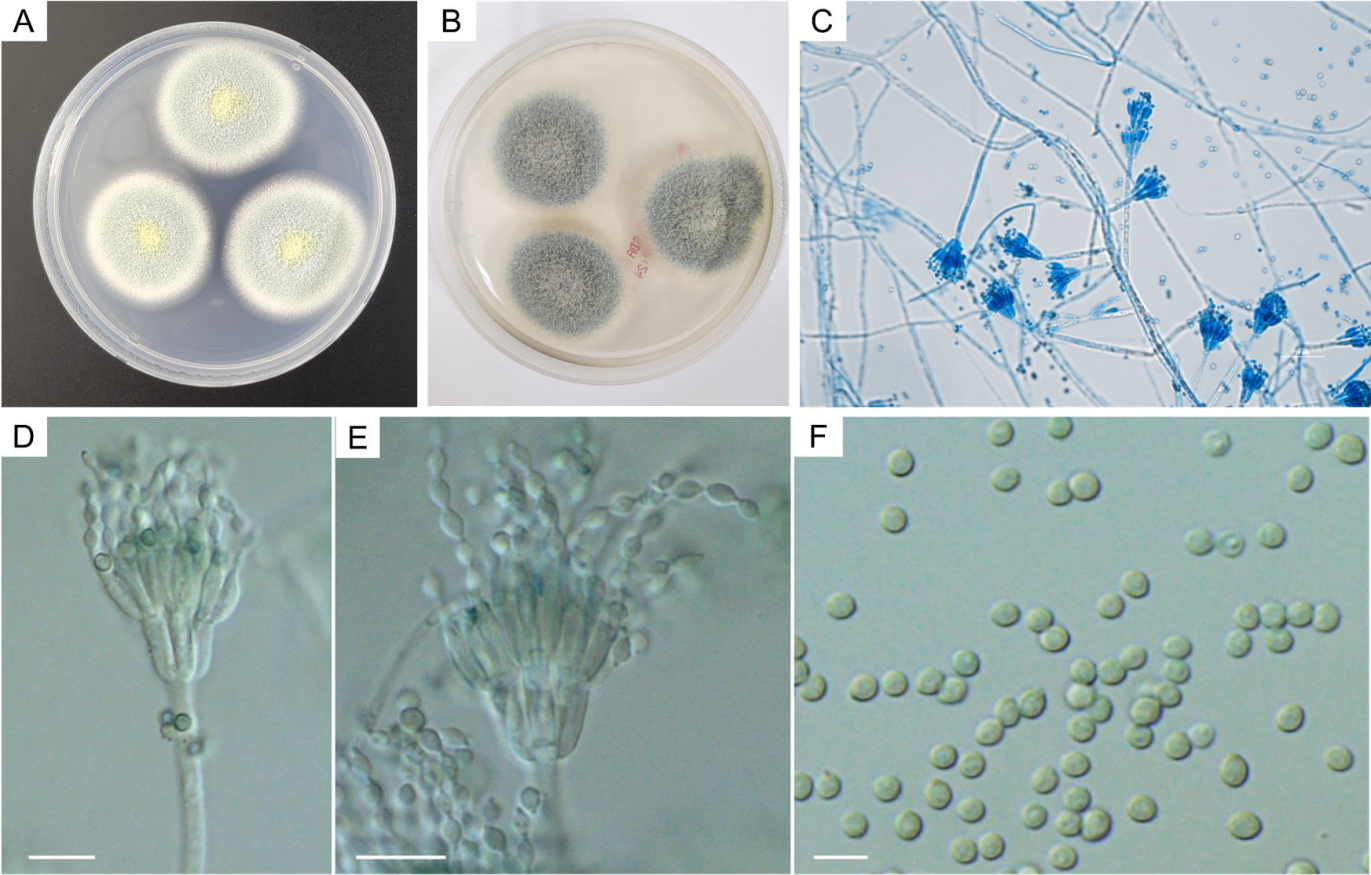
Microbiological characteristics of *T. tumuli* isolates in this case. (**A**) *T. tumuli* isolates of this case showed powder-like blue-green colonies with yellow granular center and white mycelial growth on the periphery of colonies grown on PDA and (**B**) blue-green colonies grown on SDA after 3-day incubation at 30°C. (**C**) Microscopy of this isolate revealed typical penicillium-like structures stained with lactophenol cotton blue (×200) and (**D, E**) biverticillate conidiophores and (**F**) smooth round to oval conidia prepared in 60% lactic acid (×1,000). Scale bars: D, E = 10 µm, F = 5 µm.

**Fig 3:**
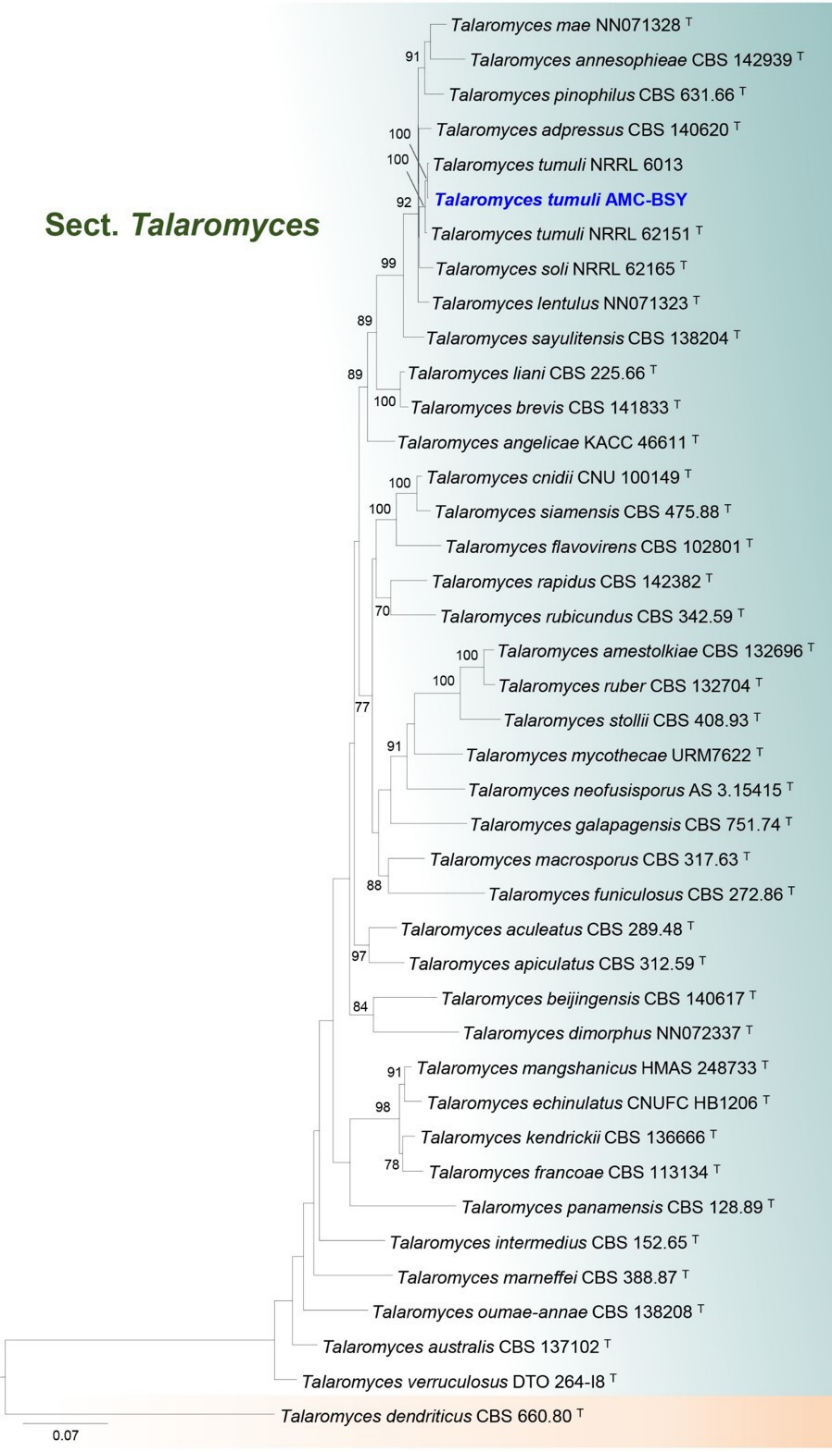
Phylogenetic tree of the combined DNA data sets of ITS, *benA*, *CaM*, and *RPB2* sequences using maximum likelihood analysis. The assembled sequence of this case (AMC-BSY strain in blue and bold letters) was clustered to *T. tumuli. Talaromyces dendriticus* CBS 660.80 was used as an outgroup in phylogenetic analyses. Ex-type strains were denoted by superscript T.

A young woman was transferred to a tertiary care hospital in Seoul, South Korea, for unresolved infective endocarditis with intermittent fever. She was receiving antimicrobial treatments to eliminate a hypermobile echogenic material of 0.26 cm in diameter on the anterior mitral leaflet found 3 months prior. She had a history of multiple hospitalizations for recurrent bacteremia caused by *Staphylococcus aureus*, *Enterobacter cloacae*, or *Klebsiella oxytoca* over the past 3 years. Peripheral leukocyte count and differential counts were all normal (Table 1). Blood culture during the first week of hospitalization was negative, and cefotaxime and ciprofloxacin were administered as empirical therapy (Table 1). A CVC was inserted through the left subclavian vein on hospital day (HD) 5 (Fig. 1). Fever persisted intermittently, but the leukocyte counts or C-reactive protein were within a normal range (Table 1). Two sets of blood cultures using peripheral blood and CVC-drawn blood were inoculated into BACTEC PLUS aerobic F and anaerobic lytic F bottles, respectively. Only CVC-drawn blood samples were detected positive from an aerobic bottle after 67 h of incubation. Gram-stained smears of the positive vials showed septated hyaline hyphae. Subculture of positive blood culture vial to potato dextrose agar (PDA) and Sabouraud dextrose agar (SDA) yielded growth after 3 days at 30°C. Green colonies grew on PDA and SDA, which showed *Penicillium*-like conidiogenesis on microscopic examination. It was not converted to yeast at incubation at 37°C. The colonies of this on Czapek yeast extract agar (CYA), malt extract agar (MEA), and yeast extract sucrose agar (YES) showed floccose to funiculose texture with a green front without diffusible pigment (9) and showed strong sporulation on YES and moderate sporulation on CYA and MEA. Conidiophores were biverticillate, and conidia were globose or subglobose to broadly ellipsoidal, and smooth-walled with a diameter of 2.5–3.0 µm (Fig. 2C through F). When the colonies on SDA were identified using the MALDI Biotyper sirius (Bruker Daltonics GmbH, Bremen, Germany) with MBT Filamentous Fungi Library version 4.0, the species with the highest score were *Talaromyces funiculosus*, *Talaromyces wortmannii*, *Talaromyces ruber,* and *Talaromyces duclauxii,* with scores of 1.63, 1.54, 1.51, and 1.46, respectively, suggesting *Talaromyces* species. However, they were not reliable for species-level identification. Sequencing of the internal ribosomal transcribed spacer (ITS) region including the 5.8S rRNA gene, D1/D2 domains of the 26S rRNA gene, and cytochrome oxidase subunit 1 (*cox 1*) revealed identities of 96.34-99.79% with various *Talaromyces* species by searching via Basic Local Alignment Search Tool (BLAST) in GenBank (https://blast.ncbi.nlm.nih.gov/Blast.cgi) or MycoBank (https://its.mycologylab.org/page/ISHAM%20Poly%20ID) (9, 10). Therefore, this was further analyzed using the sequences of partial β-tubulin (*BenA*), calmodulin (*CaM*), and the second largest subunit of RNA polymerase II (*RPB2*) genes using the primers Tub2Fd/Tub4Rd (11), CF1L/CF4 (12), and RPB2-5F/RPB2-7CR (13), respectively. The resulting sequences were edited and assembled using Lasergene SeqMan software (DNASTAR, Inc., Madison, WI, USA). These sequences were best matched to *T. tumuli* with a similarity range of 98.84%–99.57% by both BLAST and Mycobank search. Based on phylogenetic analyzes on the combined four loci (ITS, *BenA*, *CaM,* and *RPB2*), this isolate was identified to *T. tumuli*, belonging to section *Talaromyces* (Fig. 3). The sequences of ITS, D1/D2, *cox 1*, *BenA, CaM,* and *RPB2* genes of this isolate were deposited in GenBank under the accession numbers of PQ310676, PQ310677, PQ305931, PQ333008, PV915785, PV915787, and PV915786, respectively.

**TABLE 1:** Laboratory findings and antimicrobial therapy during hospital stay^*c*^

Initial blood cell counts	Results	Reference^*a*^	Lymphocyte and Ig profile	Results	Reference^*a*^
Leukocyte (×1,000/µL)	8.1	4–10	T cells (Th/Tc) (%)	80.3 (39.3/26.2)	54–82 (24–55/11–38)
Neutrophil (%)	75.1	42–75	B cells (%)	12.5	5–23
Lymphocyte (%)	17.7	25–45	NK cells (%)	6.5	5–35
Monocyte (%)	6.1	2–9	IgG (mg/dL)	1357.5	650–1600
Eosinophil (%)	0.7	1–5	IgA (mg/dL)	170.1	40–350
Basophil (%)	0.4	0–1	IgM (mg/dL)	420	50–300
Hemoglobin (g/dL)	13.4	12–16	C3 (mg/dL)	88	82–170
Platelet (×1,000/µL)	188	150–350	C4 (mg/dL)	23.4	12–36
CRP at 1st week (mg/dL)	0.1–0.68	0–0.6	CH50 (U/mL)	46.1	46.7–63.3

Hospital stay (week)	Blood cultures	β-D glucan /galactomannan	Antimicrobial therapy^*b*^		
1	No growth	NA/NA	Cefotaxime 2 g ivs q6h	Ciprofloxacin 400 mg q12h	
2	*Talaromyces*	NA/NA		Gentamicin 145 mg q8h	
3	*Talaromyces*	Negative/negative	Ceftriaxone 2 g ivs q24h	Ciprofloxacin 750 mg po bid	Voriconazole 200 mg po bid
4	*Talaromyces*	Positive/negative			
5	No growth	Positive/negative			
6	No growth	NA/NA	Cefadroxil 500 mg q8h		Itraconazole 200 mg po bid
7	No growth	Negative/negative			
8	No growth	Positive/negative			
9	No growth	Negative/NA			

^*a*^ Reference range in the institute.

^*b*^ Shaded boxes denote the weeks maintaining the antimicrobial regimens.

^*c*^ Ig, immunoglobulin; CRP, C-reactive protein; Th, helper T cells; Tc, cytotoxic T cells; NK, natural killer; NA, not available; ivs, intravenous solution; po, per os; q6h, every 6 hours; q12h, every 12 hours; q8h, every 8 hours; q24h, every 24 hours; bid, twice a day.

As a practical alternative to the reference method for antifungal susceptibility testing of molds, we performed antifungal susceptibility testing using Sensititre YeastOne YO10 assay (ThermoFisher Scientific, Waltham, MA, USA) with incubation for 48 h at 35°C in an ambient air incubator and reading the endpoints as previously reported (14). The MIC values were as follows: amphotericin B ≤0.12 µg/mL, fluconazole 128 µg/mL, voriconazole 0.5 µg/mL, posaconazole 0.12 µg/mL, itraconazole ≤0.015 µg/mL, and 5-flucytosine ≤0.06 µg/mL. CVC-drawn blood cultures were consecutively positive for the same organisms six times between HD10 and HD18. On HD18, a thrombus was found in the left internal jugular vein, and the CVC was removed, which tip culture was negative. Fever subsided, and subsequent blood cultures were negative. Voriconazole was administered from HD21 to HD34 and then, itraconazole was administered from HD35 until discharge on HD60 (Table 1). Transesophageal echocardiography and neck sonogram at 7 days after discharge revealed no abnormal findings.

## DISCUSSION

To our best knowledge, this is the first human infection caused by *T. tumuli. Penicillium-*like molds isolated from clinical specimens are often dismissed as contaminants (7). Unlike *Penicillium* species, *T. marneffei* is a true pathogen causing systemic infections such as fungemia, particularly in patients who are immunocompromised (2–4). Non-*marneffei Talaromyces* species have been reported as emerging opportunistic human pathogens (5–8). *Talaromyces* species are commonly isolated from soil, indoor environments, and food products (15, 16) and infrequently cause opportunistic infections mainly in immunocompromised hosts with malignancies or HIV infection and in those who have undergone organ transplantation or chemotherapy (17). *Talaromyces pinophilus* complex, including *T. tumuli*, is a symbiotic endophyte of plants and is saprophytic (16). Therefore, it is unusual for *T. tumuli* to cause a CVC-related bloodstream infection in a host with no known predisposing conditions. In our case, certain virulence factors of *T. tumuli* or unidentified genetic susceptibility of the host may have contributed to the development of this infection.

A biofilm-associated infection related to indwelling implants is not a common form of mold infections; however, *Fusarium* species, *Acremonium* species, and *Sporothrix* species were reported to cause CVC-related bloodstream infections in patients with cancer (18). Notably, our case was a fungemia developed in a young woman with no apparent risk factors other than indwelling CVC. Fungemia caused by non-*marneffei Talaromyces* has not been reported in immunocompetent hosts. However, she suffered from multiple episodes of spontaneous bacteremia without attributable causes, suggesting an unknown constitutional susceptibility to opportunistic infections (19). Additionally, she had certain predisposing conditions for nosocomial infections such as long-term hospitalization and antibacterial treatment (20). Except for *T. marneffei*, *Talaromyces* fungemia is rare (6). *T. pinophilus* is known to be beneficial as a wastewater treatment (16), because of the biocatalytic and biofilm-forming properties (21). The ability to produce biofilm is a well-known virulence factor to device-associated infections. Currently, data on the correlation of antifungal susceptibility and treatment outcome for non-*marneffei Talaromyces* are very limited (19). In this case, all azoles except fluconazole had excellent activity against this isolate. The exact source of *T. tumuli* is unclear; however, several blood cultures with peripheral blood samples were all negative, suggesting that the fungemia is unlikely to have originated from intravascular infections. Therefore, *T. tumuli* is an opportunistic pathogen to cause CVC-related bloodstream infections in this case.

Polyphasic species level identification using a clinical laboratory facility can identify *Talaromyces* species to a clinically relevant level. Initially, the MALDI Biotyper system is partly informative because it can differentiate species of *Penicillium* and *Talaromyces* species. At first, neither *T. tumuli* nor *T. pinophilus* complex was identified using MALDI Biotyper system. Because the first to third priority of the identification results were all *Talaromyces* species, suggesting it could be assumed to belong to *Talaromyces* genus. In addition, morphologic findings, such as the absence of thermal dimorphism, ascomata, or diffusible pigment, could rule out above-suggested non-*marneffei Talaromyces* species. The sequence analysis of ITS or D1/D2 was inconclusive, but *benA* sequencing identified *T. pinophilus* complex as the most likely among non-*marneffei Talaromyces* species. In one or more data sets of 18S rRNA, *benA*, and *cox1*, *T. flavus*, *T. funiculosus*, *T. macrosporus*, *T. marneffei*, *T. pinophilus*, or *T. purpurogenus* appeared as high-ranking matches. All the species listed belonged to section *Talaromyces* (9); further delineation was not possible because sequence data sets for the four genes evaluated in this study were not uniformly available across species using the NCBI BLAST website, or the Mycobank database. Although the ribosomal RNA sequence-based database is well-established and can be used to identify bacterial and fungal species for diagnostic purposes (22), fungal identification is more complex, as it often requires sequence analysis of regions such as *benA*, *CaM*, and *RPB2* and polyphasic approaches involving morphological and physiological characterization (9, 23). Here, sequencing of multiple genes was required to identify *T. tumuli*. In clinical laboratories, identifying non-*marneffei Talaromyces* at the species level is time-consuming and laborious and provides a limited benefit for patient care. Although morphologic findings may be helpful in ruling out most *Talaromyces* species*,* this may not be timely because it requires culture under various conditions. Therefore, early identification using MALDI-TOF MS is crucial when *Talaromyces* species cause fungemia. In the future, the MALDI-TOF MS database should be expanded to enable differentiation between *T. marneffei* and non-*marneffei Talaromyces* species. For the presumptive identification of *Penicillium/Talaromyces*, microscopic examination to observe the characteristic conidiogenesis is very useful. Furthermore, if the key phenotypic feature of *T. marneffei*, which is the production of diffusible red pigment, is absent, it can be differentiated as non-*marneffei Talaromyces* species. However, species identification of non-*marneffei Talaromyces* species is tedious and is not always crucial for guiding antifungal therapy. In cases of severe and refractory infections like this, antimicrobial susceptibility testing can be useful to guide therapy.

### Conclusion

The non-*marneffei Talaromyces* species *T. tumuli* can cause serious infections in hosts with predisposing factors, such as indwelling CVC, prolonged antimicrobial therapy, and hospitalization, and possibly constitutional susceptibility to fungal and other infections.

## Data Availability

The original contributions presented in the study are included in the article, and further inquiries can be directed to the corresponding authors. The sequences of ITS, D1/D2, *cox 1*, *BenA, CaM,* and *RPB2* genes of this isolate were deposited in GenBank under the accession numbers PQ310676, PQ310677, PQ305931, PQ333008, PV915785, PV915787, and PV915786, respectively.
